# 2216 Characterizing physician trust and healthcare-based discrimination among long-term HIV viral trajectory groups in Washington, DC

**DOI:** 10.1017/cts.2018.157

**Published:** 2018-11-21

**Authors:** Katherine G. Michel, Cuiwei Wang, Allison Doyle, Camille Robinson, Joanne M. F. Ocampo, Lakshmi Goparaju, Seble Kassaye

**Affiliations:** Georgetown – Howard Universities

## Abstract

OBJECTIVES/SPECIFIC AIMS: Discrimination within the healthcare system and physician distrust have been associated with adverse clinical outcomes for people living with HIV; however, many studies do not link these variables to biological data. We hypothesize that perceived healthcare discrimination and physician distrust associates with higher longitudinal viremia among HIV-positive women. METHODS/STUDY POPULATION: A 2006 cross-sectional survey assessed healthcare-based discrimination and physician trust in 92 HIV-positive and 46 high-risk HIV-negative women from the Washington DC Women’s Interagency HIV Study (DC-WIHS). In addition, we identified HIV viral load trajectories and demographics from the HIV-positive women who contributed≥4 semi-annual visits from 1994 to 2015. Viral suppression was defined by assay detection limits (<80 to <20 copies/mL). Group-based probability trajectory analyses grouped women based on longitudinal viral load patterns, and identified 3 groups: sustained viremia (n=32) with low-viral suppression over time, intermittent viremia (n=27) with varying suppression over time, and non-viremia (n=33) with high-longitudinal viral suppression. Ordinal logistic regression models assessed trajectory group and discrimination variables, controlling for demographics, using stepwise selection with significance level of α=0.05. RESULTS/ANTICIPATED RESULTS: Most women were African American (60%), insured at the time of visit (89%) and nonsmokers (56%). While physician trust did not differ by HIV viral trajectory group, trust was lower among HIV-negative women compared with HIV-positive women (*p*=0.03). Over 1 in 5 HIV-positive women reported discrimination in the healthcare system based on HIV status (21.3%). Report of discrimination based on drug/alcohol use was higher among HIV-negative participants (19.2% vs. 6.5%, *p*=0.01). Among women with longitudinal sustained viremia, report of discrimination based on race ethnicity (29%, *p*=0.004) and sexual orientation (15.6%, *p*=0.008) were higher than within the nonviremic and intermittent trajectory groups. DISCUSSION/SIGNIFICANCE OF IMPACT: Physician trust did not associate with increased longitudinal viral suppression among HIV-positive women in Washington, DC. Lack of physician trust among high-risk HIV-negative women could have implications for uptake of prevention methods. Reports of discrimination vary between HIV-positive and HIV-negative women in the Washington, DC area. The findings of healthcare system distrust among HIV-negative women has implications outside the realm of HIV, as this lack of trust may impact risk for other disease states among similar populations of women.

